# Red and Processed Meat and Mortality in a Low Meat Intake Population

**DOI:** 10.3390/nu11030622

**Published:** 2019-03-14

**Authors:** Saeed Mastour Alshahrani, Gary E. Fraser, Joan Sabaté, Raymond Knutsen, David Shavlik, Andrew Mashchak, Jan Irene Lloren, Michael J. Orlich

**Affiliations:** 1 School of Public Health, Loma Linda University, Loma Linda, CA 92354, USA; gfraser@llu.edu (G.E.F.); jsabate@llu.edu (J.S.); rknutsen@llu.edu (R.K.); dshavlik@llu.edu (D.S.); amashchak@llu.edu (A.M.); jclloren@llu.edu (J.I.L.); morlich@llu.edu (M.J.O.); 2 College of Applied Medical Sciences, King Khalid University, Abha 61421, Saudi Arabia; 3 School of Medicine, Loma Linda University, Loma Linda, CA 92354, USA

**Keywords:** red meat, processed meat, mortality, Adventist Health Study, cohort, Adventist

## Abstract

Associations of low-to-moderate consumption of red and processed meat with mortality would add to the evidence of possible adverse effects of these common foods. This study aims to investigate the association of red and processed meat intake with mortality. The Adventist Health Study-2 (AHS-2) is a prospective cohort study of ~96,000 Seventh-day Adventist men and women recruited in the US and Canada between 2002 and 2007. The final analytic sample after exclusions was 72,149. Cox proportional hazards regression was used and hazard ratios (HR) and confidence intervals (CI) were obtained. Diet was assessed by a validated quantitative food frequency questionnaire (FFQ), calibrated using six 24-h dietary recalls. Mortality outcome data were obtained from the National Death Index. During a mean follow-up of 11.8 years, there were 7961 total deaths, of which 2598 were Cardiovascular diseases (CVD) deaths and 1873 were cancer deaths. Unprocessed red meat was associated with risk of all-cause mortality (HR: 1.18; 95% CI: 1.07–1.31) and CVD mortality (HR: 1.26; 95% CI: 1.05–1.50). Processed meat alone was not significantly associated with risk of mortality. The combined intake of red and processed meat was associated with all-cause mortality (HR: 1.23; 95% CI: 1.11–1.36) and CVD mortality (HR: 1.34; 95% CI: 1.12–1.60). These findings suggest moderately higher risks of all-cause and CVD mortality associated with red and processed meat in a low meat intake population.

## 1. Introduction

Over the last fifty years, global meat consumption has increased in both developing and developed countries [1]. In the United States, red meat is the most commonly consumed type of meat; and processed meat represents approximately one-fourth of the total meat consumed [2]. Red and/or processed meat have been associated with higher risk of coronary heart diseases (CHD) [3], diabetes [3,4,5], and some forms of cancer [6].

Several studies have found positive associations between intake of red and/or processed meat and total, cardiovascular disease (CVD), or cancer mortality [7,8,9]. These studies were mainly conducted in populations with moderate to high levels of meat intake, leaving unanswered whether zero consumption might correlate with even lower risk. The Adventist Health Study-2 (AHS-2) has a large number of vegetarians, with even most nonvegetarians having low intakes of red and processed meat [10]. Therefore, this analysis aims to investigate the association of such low intakes of red and processed meat with all-cause, CVD, and cancer mortality.

## 2. Materials and Methods

### 2.1. Study Population

The Adventist Health Study-2 (AHS-2) has been previously described in detail [11]. It is a prospective cohort study of approximately 96,000 Seventh-day Adventist men (~35%) and women (~65%) recruited from Adventist churches in the United States and Canada between 2002 and 2007. The cohort was formed primarily to study the possible effects of diet on cancer risk. The study was approved by the Institutional Review Board of Loma Linda University, and written informed consent was obtained from each participant upon enrolment.

For this analysis, exclusions were applied: missing dietary variables (*n* = 267); bad or inadequate data (e.g., unlikely response patterns including clearly invalid and identical responses to questions in a single page) (*n* = 507); missing more than 69 responses in the dietary section (2172); Canadian residents (*n* = 4393); extreme values of total energy (e.g., <500 kcal/day or >4500 kcal/day) (*n* = 2044); missing age, sex, or race (*n* = 538); age younger than 25 years at baseline (*n* = 8); and prevalent diseases related to mortality outcomes (i.e., prevalent cancers (except nonmelanoma skin cancers) or prevalent CVD defined as previous coronary bypass, angioplasty/stent, carotid artery surgery, myocardial infarction, stroke, or transient ischemic attack (TIA); or angina pectoris or congestive heart failure treated in the past 12 months) (*n* = 13,527). The analytic sample remaining was 72,149.

### 2.2. Assessment of Exposures

AHS-2 participants were requested at enrolment to complete a quantitative food frequency questionnaire (FFQ) consisting of more than 200 food items. Unprocessed red meat intake was reported as two items in the FFQ: “hamburger, ground beef (in casserole, meatballs, etc.)” and “beef or lamb as a main dish (e.g., steak, roast, stew, and pot pies)”. Processed meat was reported as: “processed beef, lamb (e.g., sausage, salami, and bologna)” and “processed chicken or turkey (e.g., turkey bologna, and turkey ham)”. Pork was classified as processed meat because most of the pork products listed in the single pork question in the FFQ were processed (i.e., “pork (bacon, sausage, ham, chops, ribs, and lunch-meat)”). The frequency of intake ranged from “never or rarely” to “2+ per day”, and serving sizes consisted of three levels (a half serving, standard serving (3–4 oz.), and one-and-a-half servings). The assigned weights for frequency and serving size in the FFQ used in AHS-2 have been previously described [12]. The intake in grams per day was calculated using the product-sum method [12,13].

The FFQ has been validated using six 24-h dietary recalls from a representative subset of the original AHS-2 cohort [12]. The deattenuated validity correlations for all types of meat, poultry, and fish combined were 0.86 and 0.85 for Whites and Blacks, respectively. Specifically, the validity correlations for unprocessed red and processed meats were 0.74 and 0.43 for Whites, respectively, and 0.68 and 0.36 for Blacks, respectively [12].

### 2.3. Ascertainment of Outcomes

Mortality outcome data were obtained from the National Death Index and were available through December 2015. International Statistical Classification of Diseases, 10th Revision (ICD-10) codes were used to determine the underlying cause of death. Individuals who died due to unnatural causes such as motor vehicle accidents and homicides (ICD-10 letters U, V, W, X, and Y) were censored at their time of death (i.e., they were not considered as cases in the analysis of all-cause mortality). ICD-10 codes for CVD mortality ranged from I00 to I78; cancer mortality ranged from C00 to C97.

### 2.4. Assessment of Covariates

Covariates were self-reported by questionnaire at enrolment, including information on demographics, medical history (including obstetrical and gynecological history), family medical history, socioeconomic factors, lifestyle factors, and diet. Covariates were selected a priori as possible confounders (see Tables footnotes for covariates details).

### 2.5. Statistical Analysis

Time-dependent Cox-proportional hazards regression with attained age as the time variable and left truncation by age at study entry, controlling for potential confounding covariates, was used to assess the association between the consumption of red and processed meat and all-cause, CVD, and cancer mortality. We used multiple imputation, guided where possible, for missing data [14,15], in which the estimates were calculated from five imputed datasets, and then Rubin’s formula was applied to obtain the average estimates and corrected standard errors [16]. We analyzed intakes of unprocessed red and processed meats as continuous (log-transformed) variables measured in grams per day (g/day) comparing the 90th percentile of intake with zero-intake. To assess for possible common associations of unprocessed red and processed meats (which were highly correlated; *R* = 0.56), we combined both variables by summing the daily intakes from unprocessed red meat and processed meat together to create one single variable. Regression calibration of the exposures was used, where reported, to minimize the possible bias in the association estimates due to measurement error [17,18]. Only exposures of interest (red and processed meat) were calibrated, not all dietary covariates. In this procedure, a shortened version of the main questionnaire (limited to the dietary/FFQ portion), was administered to a 1000-subject (equal allocation for Whites and Blacks) subsample of the AHS-2 cohort. The subsample was approximately representative of the AHS-2 cohort in terms of gender, age, education, and vegetarian status [19]. Six 24-h dietary recalls were also collected from the subsample. From this data, a linear model was produced, regressing the intakes of red and processed meats from dietary recalls on the corresponding intakes from the food frequency questionnaire, controlling for the respective covariates present in each analytic model. The coefficients of the exposures of interest (red and processed meats) from the linear model were used to predict calibrated intake values from the intake values obtained from the FFQ of the original cohort. That is, predicted 24-h recall intake values produced by a linear model regressing intake values from the 24-h recalls against those from the FFQ, adjusted for the analytic covariates, are used in the analytic models to produce calibrated effect estimates. These calibrated intake variables were then used in the analytic models to produce calibrated hazard ratios. A 4000-round bootstrap was used to produce bias-corrected and accelerated (BCa) confidence intervals for these hazard ratios. We also conducted analyses using five categories of intake (i.e., a zero-intake group plus quartiles of consumers). Linear trends across the categories were tested by assigning the medians of intake in each quartile to all participants in that quartile and analyzing them as continuous variables. Also, we assessed the linearity of the relationship between the exposures and mortality outcomes using 4-knot restricted cubic spline regression. Dietary exposures and covariates were energy-adjusted using the residual method [20].

We tested for interaction of the exposures of interest with age, sex, and race. Other covariates were also tested for possible interactions with the exposures, and for possible interactions with each other where suspected. We separately conducted subgroup analyses by sex and race, in which we used sex- and race-specific ranges and values of intakes for the exposures. Further, we conducted separate analyses on those who reported they never smoked in order to address the effect of residual confounding by smoking in the original models. The proportional hazards assumption of the model was assessed using log(−log) plots, Schoenfeld residuals, and attained-age interaction terms; there was no violation. We also calculated population attributable risk comparing the 90th percentile of the combined intake of red and processed meat (~49 g/day) with zero-intake (assuming causality) [21]. SAS (version 9.4; SAS Institute, Inc., Cary, CA, USA) was used to perform the main analyses of the study. R (version 3.5.1 software; a Language and Environment for Statistical Computing; R Foundation for Statistical Computing, Vienna, Austria) [22] was used for multiple imputation (package “Hmisc”) [23], spline regression (package “rms”) [24], and regression calibration (package “boot”) [25].

## 3. Results

During a mean follow-up of 11.8 years, there were 7961 deaths, of which 2598 were due to CVD and 1873 were due to cancers. Compared with zero-intake subjects, those with the highest intake of unprocessed red meat were younger, less educated, and less physically active. They also had higher prevalence of current smoking, alcohol use, and slightly higher BMI. Regarding dietary characteristics, they tended to have lower intakes of cruciferous vegetables, fruits, whole grains, legumes, and nuts and seeds, and higher intakes of dairy, eggs, unprocessed poultry, and processed meat (Table 1).

Consumption of red and processed meat were associated with the risk of total, CVD, and cancer mortality among the total cohort when adjusted for age, sex, race and total energy intake (model 1, Table 2) when using all forms of the exposure (e.g., quartiles of intake versus zero-intake, *p*-trend, and both uncalibrated and calibrated 90th percentiles versus zero-intake). The associations were attenuated yet remained significant for total and CVD mortality, but not cancer mortality in the multivariable models without adjustment for other meats (model 2). In the multivariable model with mutual adjustment for other meats (model 3), participants in the 90th percentile intake of unprocessed red meat (compared with zero-intake) had a higher risk of all-cause mortality (uncalibrated, HR: 1.18; 95% CI: 1.07–1.31 and calibrated, HR: 1.51; 95% CI: 1.22–1.98; *p* < 0.001) and CVD mortality (uncalibrated, HR: 1.26; 95% CI: 1.05–1.50 and calibrated, HR: 1.64; 95% CI: 1.09–2.57; *p* = 0.017). Processed meat alone was not significantly associated with risk of mortality when adjusted for other meats. Red and processed meat (combined) were associated with higher risk of all-cause mortality (uncalibrated, HR: 1.23; 95% CI: 1.11–1.36 and calibrated, HR: 1.50; 95% CI: 1.26–1.83; *p* < 0.001) and CVD mortality (uncalibrated, HR: 1.34; 95% CI: 1.12–1.60 and calibrated, HR: 1.73; 95% CI: 1.27–2.51; *p* < 0.001) (Table 2).

**Figure 1:**
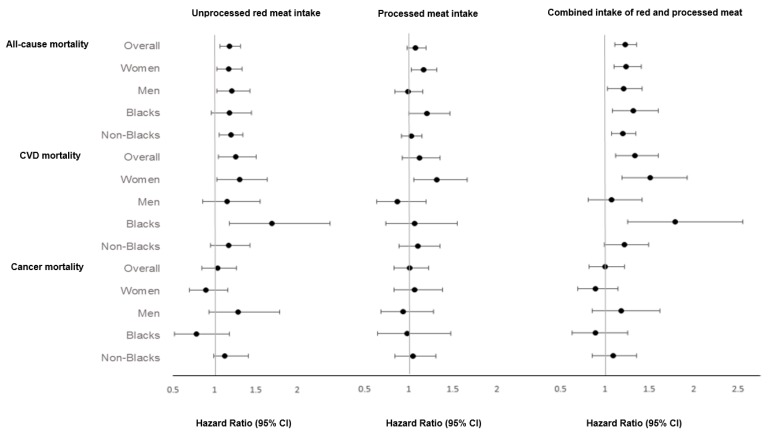
Subgroup analysis by sex and race of the association between red meat and processed meat intake and all-cause, cardiovascular, and cancer mortality. Multivariable hazard ratios for mortality comparing the 90th percentiles (sex- and race-specific values) of unprocessed red and processed meats intakes and both combined with zero-intake (90th vs. 0) were adjusted for age; sex (not in sex subgroup analysis); race (not in race subgroup analysis); marital status; education level; multivitamin use; smoking; alcohol use; exercise; sleeping hours; body mass index (BMI); diabetes mellitus; hypertension; hypercholesterolemia; aspirin use; the use of blood pressure medications for at least 2 years in the last 5 years; the use of statin for at least 2 years in the last 5 years; menopausal status in women and hormone replacement therapy (HRT) among postmenopausal women; dietary energy; and dietary variables including cruciferous vegetables, fruits, whole grain, legumes, nuts and seeds, total dairy, eggs, fish, and unprocessed poultry.

Results from subgroups are presented as forest plots, where 90th percentiles versus zero-intake contrasts were used (Figure 1). Unprocessed red meat was significantly associated with risk of all-cause mortality among women (HR: 1.17; 95% CI: 1.03–1.33), men (HR: 1.21; 95% CI: 1.03–1.43), and non-Blacks (HR: 1.20; 95% CI: 1.06–1.34), but not among Blacks (HR: 1.18; 95% CI: 0.96–1.45); with CVD mortality among women (HR: 1.30; 95% CI: 1.03–1.64), but not men (HR: 1.15; 95% CI: 0.93–1.79), and among Blacks (HR: 1.69; 95% CI: 1.18–2.40), but not non-Blacks (HR: 1.17; 95% CI: 0.95–1.43). Processed meat was associated with all-cause mortality among women (HR: 1.17; 95% CI: 1.03–1.32), but not men (HR: 0.99; 95% CI: 0.84–1.16), and among Blacks (HR: 1.21; 95% CI: 1.00–1.47), but not non-Blacks (HR: 1.03; 95% CI: 0.92–1.15); with CVD mortality among women (HR: 1.32; 95% CI: 1.06–1.66), but not among men (HR: 0.87; 95% CI: 0.64–1.20), Blacks (HR: 1.07; 95% CI: 0.74–1.55), nor non-Blacks (HR: 1.10; 95% CI: 0.89–1.36). The combined intake of red and processed meat was associated with a higher risk of all-cause mortality among all subgroups and CVD mortality among women and Blacks (Figure 1). Complete results from subgroup analyses are available as online supplemental materials (Supplementary Tables S1–S4).

We also evaluated the linear relationships of these exposures with all-cause, CVD, and cancer mortality using 4-knot restricted cubic splines (Supplementary Figure S1). Unprocessed red meat intake and combined intake of red and processed meat appeared to have more clearly linear relationships with mortality outcomes as compared to processed meat intake.

To explore the effects of residual confounding by smoking, we conducted separate analyses among participants who had never smoked (Supplementary Table S5). We report results for the 90th percentiles versus zero-intake: Unprocessed red meat intake was associated with all-cause mortality (HR: 1.16; 95% CI: 1.02–1.31) and CVD mortality (HR: 1.26; 95% CI: 1.02–1.56). Processed meat intake was associated with all-cause mortality (HR: 1.13; 95% CI: 1.00–1.26), but not with CVD mortality (HR: 1.15; 95% CI: 0.94–1.41). Combined intake of red and processed meat was associated with higher risk of all-cause ((HR: 1.22; 95% CI: 1.08–1.37) and CVD (HR: 1.34; 95% CI: 1.08–1.65) mortality among never smokers.

We calculated population attributable risk, in which we compared the 90th percentile of the combined intake of red and processed meat (~49 g/day) with zero-intake. If the relationships were causal, approximately 6.3% and 9% of total and CVD deaths, respectively, could have been prevented if those in the 90th percentile of combined intake of red and processed meat had abstained.

## 4. Discussion

In the Adventist Health Study-2 (AHS-2), we found relatively low levels of consumption of red and processed meat to be positively associated with all-cause and CVD mortality in multivariable-adjusted models, compared to zero-intake. The associations appeared to be linear (i.e., exhibiting a dose–response relationship) and of moderate strength. Stronger associations—though less precise—were observed when the exposures were calibrated suggesting that measurement error biased the uncalibrated results towards the null.

Other studies from the U.S. and Europe have found positive associations between red or processed meat consumption and all-cause and CVD mortality [7,8,9,26,27,28]. In the U.S., three large cohort studies—the Health Professionals Follow-Up Study (HPFS), the Nurses’ Health Study (NHS), and the American Association of Retired Persons (NIH-AARP) study—found associations of red and processed meat intake with all-cause, CVD, and cancer mortality among both men and women [7,8,26]. The relative risks of all-cause and CVD mortality in these studies ranged from 14 to 50% for red meat, and from 9 to 72% for processed meat. From the European Prospective Investigation into Cancer and Nutrition (EPIC) study, Rohrmann et al. found a 14% higher risk of all-cause mortality associated with red meat intake, and found 44% and 72% higher risks of all-cause and CVD mortality, respectively, associated with processed meat [9]. Furthermore, Bellavia et al., in Sweden, found higher risks of all-cause and CVD mortality by 21% and 29%, respectively, associated with red meat consumption [27]. Recently, processed meat was associated with 21% and 26% higher (relative) risks of overall and CVD mortality, respectively, in The Netherlands Cohort Study [28]. These findings appear to be compatible with ours, though they examine higher intake ranges. Meta-analyses have also found positive associations of red and/or processed meat with all-cause and/or CVD mortality [29,30,31]. In contrast, studies in Asia have generally not found associations between red meat intake and mortality [32] (except for Takata et al., who found a 18% higher risk of all-cause mortality among men) [33].

Regarding cancer mortality, the three cohorts in the US have found significant associations, in contrast to our null findings. This could be due to insufficient dietary adjustments in those studies (we adjusted for multiple dietary variables including dairy, whole grains and legumes, which they have been associated with reduced risk of some cancers) [34,35,36]; low and infrequent meat consumption in our cohort (particularly processed meat that has been linked to cancer risk, especially in populations with higher intakes) [37,38,39]; or the relative power limitations of our sample from this low cancer incidence population [40]. Lack of association for cancer mortality does not necessarily indicate no relationship to cancer incidence; for example, vegetarian dietary patterns (which are low in red and processed meat) were not at lower risk of cancer mortality compared with nonvegetarians [41], although they have been linked to lower overall cancer incidence in this cohort [42].

Previously in AHS–2, Orlich et al. found that vegetarians had a 12% reduced risk of all-cause mortality as compared to nonvegetarians [41]; this could be due to lower intake of animal foods or higher intake of plant foods among vegetarians. In our analyses, we were able to control for foods commonly consumed by vegetarians such as legumes, whole grains, and nuts and seeds [10], yet we found that highest intakes of red and processed meat were associated with an 18–51% higher risk of all-cause mortality as compared to zero-intake participants. Such findings suggest a possible effect of red and processed meat in increasing mortality after controlling for plant foods.

Some possible causal mechanisms have been proposed for the link between red and processed meat consumption and mortality. Red meat is rich in saturated fatty acids, which have been associated with dyslipidemia, particularly elevated low-density lipoprotein (LDL) [43]. High LDL levels are associated with a higher risk of atherosclerosis [44] and acute myocardial infarction [45]. Furthermore, red meat intake has been associated with increased levels of inflammatory and oxidative stress markers such as C-reactive protein (CRP) and gamma-glutamyl transferase (GGT) that have been associated with cardiovascular diseases [46]. Heme iron in red and processed red meats has been associated with higher risk of type 2 diabetes [5] and cardiovascular diseases [47,48] including myocardial infarction [49] and coronary heart disease [50]. Also, red meat is rich in L-carnitine, and recent research found that L-carnitine metabolism by intestinal microbiota elevates the level of a metabolite known as trimethylamine-N-oxide (TMAO) [51], which was linked with a higher risk of cardiovascular diseases, particularly atherosclerosis [51,52]. Additionally, large amounts of sodium are used in some meat processing [53]; high intakes of sodium are associated with elevated blood pressure, a major risk factor for CVD [54,55,56].

This study fills an important gap in the literature, in that we were able to evaluate the association of red and processed meat at low consumption levels compared to zero-intake subjects, whereas other studies have only compared risk at higher intake levels. For example, the 90th percentile of unprocessed red meat intake in our population was 46.5 g/day, which is approximately equivalent to a half serving per day—meat serving size ranges from 3 to 4 ounces (85–113 g)—in the FFQ of AHS-2. However, in HPFS and NHS, men who consumed 1.46 serving/day were compared with those who consumed 0.17 serving/day, and women who consumed 1.64 serving/day were compared with those who consumed 0.37 serving/day [7]. In NIH-AARP, the highest intakes ranged from 65.9 to 68.1 g/1000 kcal and were compared with approximately 9 g/1000 kcal as a reference group [8]. These ranges of intakes are considerably higher than those in our population. No other studies compare low-to-moderate intake with zero-intake. Another strength of this study is the use of regression calibration to minimize the effects of dietary measurement error. Although regression calibration was only used for the exposures of interest, and not for other dietary covariates, it provided less biased association estimates—though also less precise—compared to the uncalibrated results. Another strength is the relatively low prevalence of smoking and alcohol use, reducing the potential residual confounding. To further minimize the effect of confounding by smoking, we separately explored the associations among never-smoked participants, and these findings were consistent with those from the main analyses. Finally, we included detailed adjustments for multiple confounders including lifestyle factors and food groups to minimize the effect of confounding. Intakes of red and processed meat have been associated with several unfavorable lifestyle and dietary confounders including obesity, smoking, physical inactivity, and low intakes of fruits and vegetable [57]. Besides adjusting for these factors, we additionally adjusted for multiple dietary factors such as dairy, legumes, whole grains, and nuts and seeds.

An inherent limitation in this type of study is measurement error in dietary assessment. In particular, processed meat measurement by FFQ showed low validity compared to multiple recalls. This low validity correlation for processed meat may be due to the infrequent consumption of processed meat; infrequently consumed foods may be missed by dietary recalls. However, validity for unprocessed red meat was relatively high. Another limitation is the single dietary assessment, whereas dietary habits may have changed. However, a majority of subjects in the AHS-2, especially middle-aged and elderly, tend to have fairly stable dietary intakes over time [58]. Finally, the possibility of unmeasured or residual confounding remains, despite our efforts to adjust for multiple potential confounders.

## 5. Conclusions

In conclusion, we found higher all-cause and CVD mortality to be associated with relatively low intake of red and processed meat (and of unprocessed red meat in particular), compared to zero intake. While caution is appropriate in inferring causation from observational data, these results suggest possible adverse effects of red and processed meat, even with low to moderate levels of intake.

## Figures and Tables

**Table 1:** Selected characteristics of AHS-2 population by the intake of unprocessed red meat (*N* = 72,149).

	**Zero Intake**	**Quartiles of Intake g/day ^1^**			
**Characteristic**	**0**	**Q1**	**Q2**	**Q3**	**Q4**
Age (year), mean (SD) *	57.3 (14.0)	56.4 (13.8)	55.7 (13.4)	54.1 (12.7)	52.7 (12.4)
Female, *n* (%)	31,124 (66.8)	4306 (66.0)	4266 (66.9)	3989 (62.7)	3704 (58.2)
Blacks, *n* (%)	11,985 (25.7)	2089 (32.5)	2153 (33.8)	1805 (28.4)	1631 (25.6)
Married, *n* (%)	34,550 (74.1)	4409 (68.0)	4446 (69.7)	4565 (71.8)	4632 (72.7)
Graduate degree, *n* (%)	9956 (21.4)	987 (15.4)	903 (14.2)	817 (12.9)	684 (10.7)
Current multivitamin users, *n* (%)	22,462 (48.2)	3238 (50.4)	3086 (48.4)	2905 (45.7)	2790 (43.8)
Current smokers, *n* (%)	121 (0.3)	82 (1.3)	116 (1.8)	205 (3.2)	291 (4.6)
Alcohol daily users, *n* (%)	140 (0.3)	53 (0.8)	79 (1.2)	122 (1.9)	171 (2.7)
Exercise (≥150 min/week), *n* (%) ^2^	9812 (21.1)	1109 (17.2)	1118 (17.5)	975 (15.3)	896 (14.1)
Postmenopausal, *n* (%) ^3^	22,538 (72.4)	3082 (71.6)	3020 (70.8)	2744 (68.8)	2418 (65.3)
Current HRT users, *n* (%) ^4^	11,659 (37.5)	1701 (39.5)	1682 (39.4)	1572 (39.4)	1348 (36.4)
Diabetes, *n* (%)	2698 (5.8)	636 (9.9)	692 (10.9)	711 (11.2)	698 (11.0)
Hypertension, *n* (%)	8328 (17.9)	1626 (25.3)	1635 (25.6)	1601 (25.2)	1649 (25.9)
Hypercholesterolemia, *n* (%)	7309 (15.7)	1439 (22.4)	1383 (21.7)	1326 (20.9)	1399 (22.0)
Current aspirin users, *n* (%)	6264 (13.4)	1233 (19.2)	1308 (20.5)	1236 (19.4)	1312 (20.6)
BMI (kg/m^2^), mean (SD) *	26.1 (5.3)	28 (6.0)	28.7 (6.0)	29.2 (6.4)	29.9 (6.7)
Total energy (kcal), mean (SD) *	1901.3 (739.2)	1934.3 (800.7)	1853.7 (768.2)	1844.7 (777.6)	2071.2 (783.3)
Dietary variables (g/day), median, mean (SD) *					
Cruciferous vegetables	22.9 32.6 (32.1)	18.5 27.7 (29.7)	18.6 26.6 (27.4)	17.7 24.6 (24.2)	15.4 23.1 (26.7)
Fruits	306 356 (250.0)	246 302.7 (241.9)	231 281.9 (226.0)	199.1 243.2 (199.0)	155.9 200.3 (184.1)
Whole grain	162.1 185.4 (123.1)	122.1 149.4 (109.5)	107.7 139 (110.5)	92.9 120.5 (97.2)	77.5 102.3 (87.5)
Legumes	42.4 56 (48.0)	32.3 45.1 (43.6)	30.4 42 (41.9)	27.1 36.7 (36.9)	23.5 33.5 (34.6)
Nuts and seeds	20.21 25.34 (21.7)	14.06 19.80 (19.9)	12.33 17.89 (18.4)	11.55 16.33 (16.3)	9.82 14.58 (15.5)
Total dairy	46.8 115.7 (170.5)	143.6 199.1 (202.7)	163.7 215.1 (206.3)	178 228.1 (205.9)	184.1 232.9 (200.3)
Eggs	3.3 7.7 (13.3)	6.7 12.6 (17.4)	7.1 13.7 (17.6)	8.5 15.4 (18.5)	15.2 18.9 (23.7)
Unprocessed poultry	0 4.4 (13.9)	5.9 14.5 (20.5)	7.9 16.8 (21.1)	12.2 21.3 (22.4)	28.7 27.9 (23.3)
Processed meat	0 0.3 (2.5)	0.5 1.8 (5.6)	0.9 2.6 (6.0)	1.9 4 (6.9)	3.3 7.4 (12.7)
Fish	0 7.1 (17.3)	9.0 14.9 (20.7)	11.6 16 (19.5)	12.1 16.4 (18.9)	11.5 16 (18.8)

^1^ Quartiles based on percentiles of the energy-adjusted g/day intake of unprocessed red meat among the total cohort. Median quartiles (g/day) are as follows, Q1 = 4, Q2 = 9.1, Q3 = 15.6, and Q4 = 41.7. ^2^ Exercise defined as “vigorous activities, such as brisk walking, jogging, bicycling, etc., long enough or with enough intensity to work up a sweat, get your heart thumping, or get out of breath”. ^3^ Percentages were calculated among women only. ^4^ Current hormone replacement therapy users among postmenopausal women only. * SD: Standard deviation.

**Table 2:** The association between red meat and processed meat intake and all-cause, cardiovascular, and cancer mortality in the AHS-2 cohort (*N* = 72,149) ^1^.

	**Unprocessed Red Meat Intake (g/day) ^2^**							
	**Zero Intake**	**Quartiles of Intake ^3^**				***p*-trend**	**90th vs. 0 ^4^**	**90th vs. 0 ^4^**
	**0**	**Q1**	**Q2**	**Q3**	**Q4**		**Uncalibrated**	**Calibrated**
No. of participants	46,613	6431	6377	6359	6369			
**All-cause mortality**								
No. of deaths (*n* = 7961)	5376	727	673	593	592			
Model 1	1.00	1.16 (1.07–1.26)	1.27 (1.17–1.38)	1.39 (1.27–1.52)	1.58 (1.45–1.72)	<0.0001	1.56 (1.46–1.67)	2.37 (1.99–2.93)
Model 2	1.00	1.08 (0.99–1.18)	1.16 (1.06–1.27)	1.19 (1.08–1.32)	1.26 (1.14–1.39)	<0.0001	1.25 (1.15–1.36)	1.69 (1.40–2.16)
Model 3	1.00	1.06 (0.97–1.17)	1.12 (1.02–1.24)	1.14 (1.02–1.27)	1.17 (1.05–1.32)	<0.001	1.18 (1.07–1.31)	1.51 (1.22–1.98)
**CVD mortality**								
No. of deaths (*n* = 2598)	1785	250	204	178	181			
Model 1	1.00	1.24 (1.08–1.43)	1.27 (1.09–1.48)	1.41 (1.20–1.65)	1.55 (1.33–1.82)	<0.0001	1.58 (1.40–1.78)	2.41 (1.86–3.24)
Model 2	1.00	1.20 (1.03–1.39)	1.18 (1.01–1.39)	1.27 (1.07–1.50)	1.32 (1.10–1.57)	<0.001	1.36 (1.18–1.57)	2.02 (1.44–3.04)
Model 3	1.00	1.15 (0.98–1.34)	1.11 (0.93–1.32)	1.17 (0.96–1.43)	1.20 (0.97–1.47)	0.051	1.26 (1.05–1.50)	1.64 (1.09–2.57)
**Cancer mortality**								
No. of deaths (*n* = 1873)	1228	175	160	159	151			
Model 1	1.00	1.13 (0.96–1.34)	1.16 (0.98–1.37)	1.38 (1.16–1.63)	1.53 (1.29–1.82)	<0.0001	1.50 (1.31–1.72)	2.17 (1.66–2.95)
Model 2 ^5^	1.00	1.04 (0.88–1.23)	1.04 (0.87–1.24)	1.14 (0.95–1.37)	1.19 (0.95–1.37)	0.047	1.16 (0.99–1.37)	1.41 (0.98–2.05)
Model 3 ^5^	1.00	1.01 (0.85–1.21)	1.00 (0.83–1.22)	1.08 (0.88–1.33)	1.07 (0.86–1.34)	0.357	1.04 (0.85–1.27)	1.18 (0.78–1.84)
	**Processed Meat Intake (g/day) ^2^**							
No. of participants	48,127	6014	6044	6016	5948			
**All-cause mortality**								
No. of deaths (*n* = 7961)	5544	657	598	552	610			
Model 1	1.00	1.04 (0.96–1.13)	1.24 (1.14–1.35)	1.27 (1.16–1.40)	1.59 (1.46–1.74)	<0.0001	1.54 (1.43–1.66)	1.81 (1.59–2.12)
Model 2	1.00	0.98 (0.90–1.08)	1.10 (0.99–1.21)	1.09 (0.99–1.21)	1.27 (1.15–1.40)	<0.0001	1.20 (1.10–1.30)	1.38 (1.18–1.68)
Model 3	1.00	0.95 (0.86–1.05)	1.03 (0.94–1.14)	1.02 (0.91–1.13)	1.16 (1.04–1.29)	0.018	1.08 (0.98–1.20)	1.25 (0.95–1.94)
**CVD mortality**								
No. of deaths (*n* = 2598)	1821	224	199	176	178			
Model 1	1.00	1.11 (0.95–1.28)	1.32 (1.13–1.55)	1.38 (1.15–1.67)	1.53 (1.30–1.80)	<0.0001	1.54 (1.34–1.76)	1.90 (1.56–2.37)
Model 2	1.00	1.05 (0.89–1.24)	1.21 (1.02–1.44)	1.24 (1.01–1.51)	1.31 (1.09–1.57)	<0.001	1.28 (1.09–1.51)	1.68 (1.28–2.32)
Model 3	1.00	1.01 (0.84–1.21)	1.13 (0.93–1.37)	1.14 (0.92–1.42)	1.19 (0.97–1.47)	0.054	1.12 (0.93–1.36)	1.62 (0.97–3.71)
**Cancer mortality**								
No. of deaths (*n* = 1873)	1294	142	148	128	161			
Model 1	1.00	0.92 (0.77–1.10)	1.15 (0.95–1.39)	1.12 (0.92–1.36)	1.58 (1.32–1.88)	<0.0001	1.49 (1.28–1.73)	1.61 (1.28–2.04)
Model 2 ^5^	1.00	0.85 (0.71–1.02)	1.00 (0.82–1.21)	0.94 (0.77–1.15)	1.19 (0.98–1.45)	0.229	1.12 (0.94–1.33)	1.09 (0.79–1.50)
Model 3 ^5^	1.00	0.80 (0.66–0.96)	0.93 (0.75–1.14)	0.86 (0.69–1.06)	1.06 (0.86–1.32)	0.994	1.01 (0.83–1.23)	0.74 (0.32–1.38)
	**Combined intake of red and processed meat (g/day) ^2^**							
No. of participants	40,287	7966	7965	7966	7965			
**All-cause mortality**								
No. of deaths (*n* = 7961)	4706	860	890	752	753			
Model 1	1.00	1.07 (0.99–1.15)	1.20 (1.11–1.30)	1.35 (1.24–1.46)	1.60 (1.47–1.73)	<0.0001	1.55 (1.45–1.65)	1.86 (1.68–2.09)
Model 2	1.00	1.03 (0.95–1.12)	1.11 (1.02–1.21)	1.18 (1.08–1.29)	1.27 (1.16–1.40)	<0.0001	1.25 (1.16–1.36)	1.44 (1.27–1.65)
Model 3 ^6^	1.00	1.02 (0.93–1.12)	1.09 (0.99–1.21)	1.17 (1.04–1.30)	1.25 (1.12–1.40)	<0.0001	1.23 (1.11–1.36)	1.50 (1.26–1.83)
**CVD mortality**								
No. of deaths (*n* = 2598)	1564	291	290	230	223			
Model 1	1.00	1.11 (0.96–1.27)	1.27 (1.11–1.45)	1.38 (1.20–1.58)	1.56 (1.35–1.80)	<0.0001	1.57 (1.40–1.77)	1.90 (1.59–2.26)
Model 2	1.00	1.09 (0.93–1.27)	1.21 (1.04–1.40)	1.25 (1.07–1.47)	1.33 (1.12–1.57)	<0.0001	1.37 (1.19–1.58)	1.66 (1.32–2.12)
Model 3 ^6^	1.00	1.08 (0.90–1.28)	1.18 (0.99–1.40)	1.21 (1.00–1.47)	1.29 (1.06–1.58)	0.005	1.34 (1.12–1.60)	1.73 (1.27–2.51)
**Cancer mortality**								
No. of deaths (*n* = 1873)	1080	196	206	194	197			
Model 1	1.00	1.00 (0.85–1.18)	1.12 (0.96–1.30)	1.25 (1.05–1.48)	1.57 (1.35–1.84)	<0.0001	1.48 (1.29–1.69)	1.73 (1.44–2.09)
Model 2 ^5^	1.00	0.94 (0.80–1.11)	1.00 (0.85–1.18)	1.05 (0.88–1.26)	1.19 (1.00–1.43)	0.103	1.14 (0.97–1.34)	1.25 (0.97–1.60)
Model 3 ^5^^,6^	1.00	0.88 (0.73–1.05)	0.92 (0.77–1.10)	0.97 (0.78–1.20)	1.07 (0.87–1.32)	0.604	1.00 (0.82–1.22)	1.02 (0.70–1.42)

^1^ Data are given as hazard ratio (95% confidence interval). ^2^ Values based on energy-adjusted variables. ^3^ Quartiles are based on percentiles of the energy-adjusted g/day intake of total cohort. Median quartiles (g/day) are as follows, for unprocessed red meat, Q1 = 4, Q2 = 9.1, Q3 = 15.6, Q4 = 41.7; for processed meat, Q1 = 0.7, Q2 = 1.4, Q3 = 3.3, Q4 = 9.4; and for combined intake of red and processed meats, Q1 = 1.4, Q2 = 7.3, Q3 = 15.4, Q4 = 42.8. Model 1 adjusted for age (attained age as time variable), sex (male and female), race (Black and non-Black), and total energy intake (continuous). Model 2 adjusted for age (attained age as time variable), sex (male and female), race (Black and non-Black), total energy intake (continuous), marital status (married/common-law and single/widowed/divorced/separated), educational level (up to high school graduate, trade school/some college/associate degree, bachelor degree, and graduate degree), multivitamin use (current use), smoking status (current smoker, quit <1 year, quit 1–4 years, quit 5–9 years, quit 10–19 years, quit 20–29 years, quit ≥30 years, and never smoked), alcohol use (none, rarely, monthly, weekly, and daily), exercise (none, ≤20 min/week, 21–60 min/week, 61–150 min/week, and ≥151 min/week), sleep (≤4 h/night, 5–8 h/night, and ≥9 h/night), body mass index (<18.5, 18.5–24.9, 25.0–29.9, and ≥30.0), aspirin use (yes/no: used weekly for at least two years in the last five years), having ever been diagnosed with or received treatment in the last 12 months for diabetes (yes/no), having been diagnosed in the last 5 years with or received treatment in the last 12 months for hypertension or hypercholesterolemia (yes/no), the use of statin for at least 2 years in the last 5 years, the use of blood pressure medications for at least 2 years in the last 5 years (yes/no), and dietary variables (each variable has 5 levels in g/day) as follows. Cruciferous vegetables (Quintiles: <9.6, 9.6–16.7, >16.7–26.1, >26.1–45.2, >45.2), fruits (Quintiles: <130, 130–224.4, >224.4–322, >322–464.2, >464.2), whole grain (Quintiles: <65, 65–109.9, >109.9–170.3, >170.3–252.2, >252.2), legumes (Quintiles: <17, 17–29.7, >29.7–45.9, >45.9–77.1, >77.1), nuts and seeds (Quintiles: <6.4, 6.4–12.8, >12.8–21.6, >21.6–35.1, >35.1), total dairy (0 intake, quartiles of intake: >0–36, >36–108.1, >108.1–240.9, >240.9), eggs (0 intake, quartiles of intake: >0–3.6, >3.6–7.3, >7.3–20.1, >20.1); and in women, the model also adjusted for menopausal status (premenopausal, postmenopausal), and hormone therapy (in postmenopausal women) (not taking hormone therapy, taking hormone therapy). Model 3: In addition to covariates in model 2, also adjusted for other meat variables such as fish (0 intake, quartiles of intake: >0–7, >7–12.6, >12.6–21.4, >21.4), and unprocessed poultry (0 intake, quartiles of intake: >0–4.8, >4.8–10.4, >10.4–32.5, >32.5). Also, for model 3 in unprocessed red meat, processed meat was adjusted for (0 intake and quartiles of intake) and vice versa. ^4^ Models in these analyses are correspondents to models 1, 2, and 3, except energy-adjusted log-transformed continuous dietary variables were used instead of five-level adjustment (90th percentile for unprocessed red meat: 46.5 g/day; for processed meat: 11 g/day; and for combined intake of red and processed meats: 49.1 g/day). ^5^ Also adjusted for previous screening for colon, prostate, or breast cancers during the last four years. ^6^ Model 3 here did not adjust for either unprocessed red meat or processed meat, but rather both were combined and used as one exposure variable.

## Supplementary Materials

The following are available online at https://www.mdpi.com/2072-6643/11/3/622/s1. Table S1: The association between red and processed meat intake and all-cause, cardiovascular and cancer mortality among women, Table S2: The association between red and processed meat intake and all-cause, cardiovascular and cancer mortality among men, Table S3: The association between red and processed meat intake and all-cause, cardiovascular and cancer mortality among Blacks, Table S4: The association between red and processed meat intake and all-cause, cardiovascular and cancer mortality among non-Blacks, Table S5: The association between red and processed meat intake and all-cause, cardiovascular, and cancer mortality among participants who never smoked, Figure S1: Dose–response relationships of red and processed meats with the risk of all-cause, CVD, and cancer mortality in the AHS-2 cohort.
