# Supplementary material for: Red and Processed Meat and Mortality in a Low Meat Intake Population
Source: Nutrients. 2019 Mar 14;11(3):622. doi: 10.3390/nu11030622 (PMC6470727; doi:10.3390/nu11030622)
Supplement: Supplementary file 1 [file nutrients-11-00622-s001.pdf]

Supplementary Materials

Red and processed meat and mortality in a low-meat-intake population

Saeed Mastour Alshahrani; Gary E. Fraser; Joan Sabaté; Raymond Knutsen; David Shavlik; Andrew Mashchak; Jan Irene Lloren; Michael J. Orlich

CONTENTS:

Table S1. The association between red and processed meat intake and all-cause, cardiovascular and cancer mortality among women.

Table S2. The association between red and processed meat intake and all-cause, cardiovascular and cancer mortality among men.

Table S3. The association between red and processed meat intake and all-cause, cardiovascular and cancer mortality among Blacks.

Table S4. The association between red and processed meat intake and all-cause, cardiovascular and cancer mortality among non-Blacks.

Table S5. The association between red and processed meat intake and all-cause, cardiovascular and cancer mortality among participants who never smoked.

Figure S1. Dose-response relationships of red and processed meats with the risk of all-cause, CVD and cancer mortality in the AHS-2 cohort.

Table S1. The association between red and processed meat intake and all-cause, cardiovascular and cancer mortality among women (N=47,389) [a]

	Unprocessed red meat intake (g/d) [b]						
	Zero intake	Quartiles of intake [c]				P-trend	90th vs 0 [d]
	0	Q1	Q2	Q3	Q4		
No. of participants	31,124	4,071	4,078	4,056	4,060		
**All–cause mortality**							
No. of deaths (N=4,870)	3,362	422	400	341	345		
Model 1	1.00	1.11 (1.00–1.24)	1.26 (1.14–1.41)	1.38 (1.23–1.55)	1.57 (1.40–1.76)	<.0001	1.53 (1.40–1.67)
Model 2	1.00	1.07 (0.95–1.20)	1.17 (1.04–1.31)	1.23 (1.09–1.39)	1.32 (1.17–1.50)	<.0001	1.29 (1.16–1.43)
Model 3	1.00	1.03 (0.92–1.17)	1.11 (0.98–1.26)	1.14 (0.99–1.31)	1.20 (1.04–1.38)	.004	1.17 (1.03–1.33)
**CVD–mortality**							
No. of deaths (N=1,617)	1,143	137	122	111	104		
Model 1	1.00	1.08 (0.90–1.30)	1.24 (1.03–1.50)	1.54 (1.26–1.88)	1.55 (1.26–1.91)	<.0001	1.57 (1.34–1.84)
Model 2	1.00	1.10 (0.91–1.35)	1.20 (0.98–1.48)	1.49 (1.20–1.86)	1.42 (1.13–1.79)	<.0001	1.48 (1.22–1.78)
Model 3	1.00	1.08 (0.88–1.33)	1.14 (0.90–1.43)	1.36 (1.06–1.75)	1.26 (0.97–1.63)	.016	1.30 (1.03–1.64)
**Cancer mortality**							
No. of deaths (N=1,141)	775	105	90	88	83		
Model 1	1.00	1.12 (0.91–1.38)	1.07 (0.86–1.34)	1.23 (0.98–1.54)	1.36 (1.08–1.72)	.003	1.33 (1.11–1.59)
Model 2 [e]	1.00	1.05 (0.84–1.30)	0.97 (0.77–1.22)	1.05 (0.83–1.34)	1.12 (0.86–1.44)	.488	1.07 (0.87–1.32)
Model 3 [e]	1.00	0.97 (0.76–1.22)	0.88 (0.69–1.13)	0.94 (0.72–1.23)	0.96 (0.72–1.29)	.685	0.90 (0.70–1.16)
	**Processed meat intake (g/d) [b]**						
	**Zero intake**	**Quartiles of intake [c]**				**P-trend**	**90th vs 0 [d]**
	**0**	**Q1**	**Q2**	**Q3**	**Q4**		
No. of participants	32,167	3,835	3,803	3,815	3,769		
**All–cause mortality**							
No. of deaths (N=4,870)	3,439	392	337	333	369		
Model 1	1.00	1.02 (0.91–1.14)	1.22 (1.08–1.37)	1.31 (1.16–1.48)	1.58 (1.40–1.78)	<.0001	1.57 (1.42–1.73)
Model 2	1.00	0.97 (0.87–1.10)	1.08 (0.96–1.22)	1.17 (1.03–1.34)	1.33 (1.16–1.51)	<.0001	1.29 (1.16–1.44)
Model 3	1.00	0.94 (0.83–1.06)	1.02 (0.90–1.16)	1.08 (0.94–1.24)	1.19 (1.04–1.37)	.019	1.17 (1.03–1.32)

**CVD–mortality**							
No. of deaths (N=1,617)	1,144	136	112	101	124		
Model 1	1.00	1.03 (0.85–1.25)	1.27 (1.03–1.57)	1.37 (1.07–1.75)	1.64 (1.34–2.00)	<.0001	1.63 (1.37–1.94)
Model 2	1.00	1.02 (0.83–1.25)	1.21 (0.96–1.52)	1.35 (1.04–1.75)	1.51 (1.21–1.89)	<.0001	1.50 (1.23–1.83)
Model 3	1.00	1.00 (0.80–1.25)	1.14 (0.89–1.47)	1.25 (0.95–1.65)	1.34 (1.05–1.72)	.010	1.32 (1.06–1.66)
**Cancer mortality**							
No. of deaths (N=1,141)	810	79	86	73	93		
Model 1	1.00	0.85 (0.66–1.09)	1.18 (0.93–1.49)	1.05 (0.81–1.35)	1.47 (1.16–1.86)	.004	1.42 (1.16–1.73)
Model 2 [e]	1.00	0.80 (0.61–1.04)	1.04 (0.82–1.33)	0.91 (0.70–1.18)	1.22 (0.94–1.57)	.388	1.15 (0.91–1.44)
Model 3 [e]	1.00	0.73 (0.56–0.96)	0.97 (0.76–1.25)	0.84 (0.64–1.12)	1.11 (0.84–1.46)	.940	1.07 (0.83–1.38)

Combined intake of red and processed meat (g/d) [b]

	Zero intake	Quartiles of intake [c]				P-trend	90th vs 0 [d]
	**0**	**Q1**	**Q2**	**Q3**	**Q4**		
No. of participants	26,843	5,136	5,137	5,137	5,136		
**All–cause mortality**							
No. of deaths (N=4,870)	2,940	498	525	466	441		
Model 1	1.00	1.04 (0.93–1.15)	1.15 (1.04–1.27)	1.36 (1.22–1.50)	1.59 (1.44–1.76)	<.0001	1.55 (1.42–1.69)
Model 2	1.00	1.02 (0.91–1.14)	1.09 (0.98–1.22)	1.21 (1.09–1.36)	1.35 (1.20–1.52)	<.0001	1.30 (1.16–1.17)
Model 3 [f]	1.00	1.00 (0.89–1.12)	1.06 (0.93–1.20)	1.17 (1.03–1.34)	1.29 (1.13–1.48)	<.001	1.24 (1.10–1.41)
**CVD–mortality**							
No. of deaths (N=1,617)	996	171	167	146	137		
Model 1	1.00	1.03 (0.87–1.22)	1.13 (0.96–1.35)	1.43 (1.19–1.72)	1.63 (1.35–1.96)	<.0001	1.58 (1.36–1.84)
Model 2	1.00	1.06 (0.88–1.27)	1.15 (0.95–1.39)	1.39 (1.13–1.71)	1.53 (1.23–1.90)	<.0001	1.52 (1.26–1.84)
Model 3 [f]	1.00	1.08 (0.88–1.33)	1.18 (0.94–1.47)	1.39 (1.08–1.79)	1.51 (1.16–1.97)	.001	1.51 (1.19–1.93)
**Cancer mortality**							
No. of deaths (N=1,141)	679	113	124	118	107		
Model 1	1.00	0.98 (0.80–1.21)	1.07 (0.88–1.30)	1.21 (0.97–1.51)	1.37 (1.11–1.68)	.002	1.33 (1.12–1.58)
Model 2 [e]	1.00	0.94 (0.76–1.16)	0.98 (0.79–1.21)	1.04 (0.82–1.32)	1.10 (0.87–1.40)	.477	1.07 (0.87–1.32)
Model 3 [e f]	1.00	0.83 (0.66–1.05)	0.85 (0.66–1.08)	0.90 (0.68–1.19)	0.94 (0.71–1.23)	.583	0.89 (0.69–1.15)

[a] Data are given as hazard ratio (95% confidence interval).

[b] Values based on energy-adjusted variable.

[c] Quartiles based on percentiles of the energy-adjusted g/d intake among women. Median quartiles (g/d) are as follow: for unprocessed red meat, Q1=4, Q2=9.1, Q3=15.6, Q4=40.6; for processed meat, Q1=0.6, Q2=1.3, Q3=2.9, Q4=8.2; and for combined intake of red and processed meats, Q1=1.1, Q2=6.8, Q3=14.2, Q4=39.1.

Model 1 adjusted for age (attained age as time variable), race (black and nonblack), and total energy intake (continuous).

Model 2 adjusted for age (attained age as time variable), race (black, nonblack), total energy intake (continuous), marital status (married/common-law and single/widowed/divorced/separated), educational level (up to high school graduate, trade school/some college/associate degree, bachelor degree, and graduate degree), multivitamin use (current use), smoking status (current smoker, quit <1 year, quit 1–4 years, quit 5–9 years, quit 10–19 years, quit 20–29 years, quit ≥30 years, and never smoked), alcohol use (none, rarely, monthly, weekly, and daily), exercise (none, ≤20 min/wk, 21–60 min/wk, 61–150 min/wk, and ≥151 min/wk), and sleep (≤4 h/night, 5–8 h/night, and ≥9 h/night), body mass index (<18.5, 18.5–24.9, 25.0–29.9, or ≥30.0), aspirin use (used weekly for at least two years in the last five years), having been ever diagnosed with or received treatment in the last 12 months for diabetes, having been diagnosed in the last 5 years with or received treatment in the last 12 months for hypertension or hypercholesterolemia, the use of statin or blood pressure medications for at least 2 years in the last 5 years, menopausal status (premenopausal, postmenopausal), hormone therapy (in postmenopausal women) (not taking hormone therapy, taking hormone therapy), and dietary variables (each variable has 5 levels in g/d) as following: cruciferous vegetables (Quintiles: <9.6, 9.6–16.7, >16.7–26.1, >26.1–45.2, >45.2), fruits (Quintiles: <130, 130–224.4, >224.4–322, >322–464.2, >464.2), whole grain (Quintiles: <65, 65–109.9, >109.9–170.3, >170.3–252.2, >252.2), legumes (Quintiles: <17, 17–29.7, >29.7–45.9, >45.9–77.1, >77.1), nuts and seeds (Quintiles: <6.4, 6.4–12.8, >12.8–21.6, >21.6–35.1, >35.1), total dairy (0 intake, quartiles of intake: >0–36, >36–108.1, >108.1–240.9, >240.9), eggs (0 intake, quartiles of intake: >0–3.6, >3.6–7.3, >7.3–20.1, >20.1).
Model 3: In addition to covariates in model 2, also adjusted for other meat variables such as fish (0 intake, quartiles of intake: >0–7, >7–12.6, >12.6–21.4, >21.4) and unprocessed poultry (0 intake, quartiles of intake: >0–4.8, >4.8–10.4, >10.4–32.5, >32.5). Also, for model 3 in unprocessed red meat, processed meat was adjusted for (0 intake and quartiles of intake) and vice versa.
[d] Models in theses analyses are correspondents to models 1, 2, and 3, except energy-adjusted log-transformed continuous dietary variables were used instead of five-level adjustment (90th percentile for unprocessed red meat: 43.2 g/d; for processed meat: 9.7 g/d; and for combined intake of red and processed meats: 48.4 g/d).
[e] Also adjusted for previous screening for colon or breast cancers during the last four years.
[f] Model 3 here did not adjust for either unprocessed red meat or processed meat, but rather both were combined and used as one exposure variable.

Table S2. The association between red and processed meat intake and all-cause, cardiovascular and cancer mortality among men (N=24,760) [a]

	Unprocessed red meat intake (g/d) [b]						
	Zero intake	Quartiles of intake [c]				P-trend	90th vs 0 [d]
	0	Q1	Q2	Q3	Q4		
No. of participants	15,489	2,344	2,316	2,303	2,308		
**All–cause mortality**							
No. of deaths (N=3,091)	2,014	320	278	243	236		
Model 1	1.00	1.25 (1.11–1.41)	1.34 (1.17–1.52)	1.30 (1.14–1.49)	1.65 (1.44–1.89)	<.0001	1.60 (1.44–1.78)
Model 2	1.00	1.12 (0.98–1.27)	1.15 (1.00–1.32)	1.07 (0.92–1.24)	1.22 (1.04–1.43)	.017	1.19 (1.05–1.36)
Model 3	1.00	1.11 (0.96–1.28)	1.14 (0.97–1.33)	1.06 (0.89–1.26)	1.21 (1.00–1.45)	.066	1.21 (1.03–1.43)
**CVD–mortality**							
No. of deaths (N=981)	642	116	84	69	70		
Model 1	1.00	1.47 (1.20–1.79)	1.38 (1.08–1.76)	1.20 (0.93–1.55)	1.59 (1.24–2.05)	<.0001	1.58 (1.30–1.91)
Model 2	1.00	1.31 (1.06–1.63)	1.20 (0.92–1.55)	0.98 (0.75–1.30)	1.19 (0.89–1.59)	.319	1.19 (0.94–1.49)
Model 3	1.00	1.19 (0.94–1.50)	1.05 (0.78–1.41)	0.89 (0.65–1.23)	1.13 (0.81–1.58)	.903	1.15 (0.86–1.55)
**Cancer mortality**							
No. of deaths (N=732)	453	75	71	71	62		
Model 1	1.00	1.23 (0.96–1.58)	1.30 (0.99–1.71)	1.59 (1.23–2.05)	1.77 (1.35–2.33)	<.0001	1.79 (1.45–2.21)
Model 2 [e]	1.00	1.08 (0.83–1.41)	1.10 (0.83–1.46)	1.25 (0.94–1.67)	1.23 (0.90–1.68)	.088	1.29 (0.99–1.68)
Model 3 [e]	1.00	1.16 (0.87–1.54)	1.17 (0.86–1.60)	1.31 (0.94–1.83)	1.23 (0.85–1.77)	.117	1.29 (0.93–1.79)
	**Processed meat intake (g/d) [b]**						
	**Zero intake**	**Quartiles of intake [c]**				**P-trend**	**90th vs 0 [d]**
	**0**	**Q1**	**Q2**	**Q3**	**Q4**		
No. of participants	15,960	2,196	2,227	2,196	2,181		
**All–cause mortality**							
No. of deaths (N=3,091)	2,105	267	263	232	224		
Model 1	1.00	1.10 (0.96–1.25)	1.25 (1.09–1.43)	1.32 (1.14–1.53)	1.51 (1.31–1.74)	<.0001	1.50 (1.33–1.70)
Model 2	1.00	1.00 (0.87–1.15)	1.07 (0.93–1.23)	1.05 (0.90–1.23)	1.11 (0.94–1.31)	.184	1.08 (0.94–1.24)
Model 3	1.00	0.97 (0.83–1.12)	1.01 (0.86–1.18)	1.00 (0.83–1.19)	1.04 (0.87–1.26)	.732	0.99 (0.84–1.16)

**CVD–mortality**							
No. of deaths (N=981)	677	87	91	74	52		
Model 1	1.00	1.21 (0.95–1.56)	1.46 (1.15–1.85)	1.44 (1.11–1.85)	1.24 (0.92–1.66)	<.001	1.39 (1.12–1.73)
Model 2	1.00	1.10 (0.84–1.44)	1.21 (0.94–1.57)	1.12 (0.85–1.49)	0.95 (0.68–1.32)	.576	1.01 (0.78–1.32)
Model 3	1.00	0.97 (0.73–1.29)	1.08 (0.81–1.45)	1.02 (0.75–1.38)	0.88 (0.60–1.29)	.821	0.87 (0.64–1.20)
**Cancer mortality**							
No. of deaths (N=732)	484	59	69	56	64		
Model 1	1.00	0.96 (0.70–1.30)	1.26 (0.95–1.66)	1.25 (0.92–1.69)	1.70 (1.29–2.22)	<.001	1.61 (1.28–2.03)
Model 2 [e]	1.00	0.84 (0.61–1.15)	1.05 (0.78–1.41)	0.94 (0.69–1.30)	1.14 (0.84–1.55)	.624	1.08 (0.83–1.42)
Model 3 [e]	1.00	0.83 (0.59–1.16)	0.99 (0.71–1.37)	0.86 (0.61–1.22)	1.00 (0.71–1.42)	.796	0.94 (0.68–1.28)

	Combined intake of red and processed meat (g/d) [b]						
	Zero intake	Quartiles of intake [c]				P-trend	90th vs 0 [d]
	0	Q1	Q2	Q3	Q4		
No. of participants	13,444	2,829	2,829	2,829	2,829		
**All–cause mortality**							
No. of deaths (N=3,091)	1,766	371	354	297	303		
Model 1	1.00	1.13 (1.01–1.27)	1.25 (1.11–1.41)	1.33 (1.16–1.51)	1.66 (1.47–1.88)	<.0001	1.56 (1.40–1.73)
Model 2	1.00	1.07 (0.94–1.21)	1.10 (0.96–1.25)	1.10 (0.95–1.27)	1.23 (1.06–1.43)	.010	1.19 (1.04–1.36)
Model 3 [f]	1.00	1.07 (0.92–1.25)	1.10 (0.94–1.27)	1.12 (0.93–1.34)	1.26 (1.05–1.51)	.021	1.21 (1.03–1.42)
**CVD–mortality**							
No. of deaths (N=981)	568	126	116	81	90		
Model 1	1.00	1.30 (1.03–1.65)	1.39 (1.13–1.71)	1.23 (0.97–1.56)	1.60 (1.27–2.01)	<.0001	1.57 (1.30–1.89)
Model 2	1.00	1.21 (0.94–1.56)	1.23 (0.98–1.54)	1.02 (0.78–1.33)	1.21 (0.92–1.60)	.218	1.17 (0.93–1.48)
Model 3 [f]	1.00	1.08 (0.80–1.47)	1.06 (0.80–1.40)	0.88 (0.63–1.21)	1.10 (0.79–1.53)	.998	1.07 (0.81–1.42)
**Cancer mortality**							
No. of deaths (N=732)	401	82	81	84	84		
Model 1	1.00	1.02 (0.78–1.34)	1.19 (0.91–1.55)	1.41 (1.08–1.83)	1.90 (1.49–2.41)	<.0001	1.74 (1.41–2.15)
Model 2 [e]	1.00	0.93 (0.70–1.23)	1.00 (0.75–1.33)	1.13 (0.85–1.49)	1.28 (0.95–1.71)	.116	1.23 (0.95–1.61)
Model 3 [e f]	1.00	0.96 (0.71–1.31)	1.03 (0.75–1.42)	1.18 (0.84–1.65)	1.28 (0.91–1.79)	.111	1.18 (0.86–1.62)

[a] Data are given as hazard ratio (95% confidence interval).

[b] Values based on energy-adjusted variable.

[c] Quartiles based on percentiles of the energy-adjusted g/d intake among men. Median quartiles (g/d) are as follow: for unprocessed red meat, Q1=4, Q2=9.1, Q3=15.5, Q4=43.6; for processed meat, Q1=0.7, Q2=1.6, Q3=4, Q4=11.3; and for combined intake of red and processed meats, Q1=1.7, Q2=8.6, Q3=17.9, Q4=50.2.

Model 1 adjusted for age (attained age as time variable), race (black, nonblack), and total energy intake (continuous).

Model 2 adjusted for age (attained age as time variable), race (black, nonblack), total energy intake (continuous), marital status (married/common-law and single/widowed/divorced/separated), educational level (up to high school graduate, trade school/some college/associate degree, bachelor degree, and graduate degree), multivitamin use (current use), smoking status (current smoker, quit <1 year, quit 1–4 years, quit 5–9 years, quit 10–19 years, quit 20–29 years, quit ≥30 years, and never smoked), alcohol use (none, rarely, monthly, weekly, and daily), exercise (none, ≤20 min/wk, 21–60 min/wk, 61–150 min/wk, and ≥151 min/wk), and sleep (≤4 h/night, 5–8 h/night, and ≥9 h/night), body mass index (<18.5, 18.5–24.9, 25.0–29.9, or ≥30.0), aspirin use (used weekly for at least two years in the last five years), having been ever diagnosed with or received treatment in the last 12 months for diabetes, having been diagnosed in the last 5 years with or received treatment in the last 12 months for hypertension or hypercholesterolemia, the use of statin or blood pressure medications for at least 2 years in the last 5 years, and dietary variables (each variable has 5 levels in g/d) as following: cruciferous vegetables (Quintiles: <9.6, 9.6–16.7, >16.7–26.1, >26.1–45.2, >45.2), fruits (Quintiles: <130, 130–224.4, >224.4–322, >322–464.2, >464.2), whole grain (Quintiles: <65, 65–109.9, >109.9–170.3, >170.3–252.2, >252.2), legumes (Quintiles: <17, 17–29.7, >29.7–45.9, >45.9–77.1, >77.1), nuts and seeds (Quintiles: <6.4, 6.4–12.8, >12.8–21.6, >21.6–35.1, >35.1), total dairy (0 intake, quartiles of intake: >0–36, >36–108.1, >108.1–240.9, >240.9), eggs (0 intake, quartiles of intake: >0–3.6, >3.6–7.3, >7.3–20.1, >20.1).

Model 3: In addition to covariates in model 2, also adjusted for other meat variables such as fish (0 intake, quartiles of intake: >0–7, >7–12.6, >12.6–21.4, >21.4) and unprocessed poultry (0 intake, quartiles of intake: >0–4.8, >4.8–10.4, >10.4–32.5, >32.5). Also, for model 3 in unprocessed red meat, processed meat was adjusted for (0 intake and quartiles of intake) and vice versa.

[d] Models in theses analyses are correspondents to models 1, 2, and 3, except energy-adjusted log-transformed continuous dietary variables were used instead of five-level adjustment (90th percentile for unprocessed red meat: 52.9 g/d; for processed meat: 13 g/d; and for combined intake of red and processed meats: 56.7 g/d).

[e] Also adjusted for previous screening for colon or prostate cancers during the last four years.

[f] Model 3 here did not adjust for either unprocessed red meat or processed meat, but rather both were combined and used as one exposure variable.

Table S3. The association between red and processed meat intake and all-cause, cardiovascular and cancer mortality among Blacks (N=19,663) [a]

	Unprocessed red meat intake (g/d) [b]						
	**Zero intake**	**Quartiles of intake [c]**				**P-trend**	**90th vs 0 [d]**
	**0**	**Q1**	**Q2**	**Q3**	**Q4**		
No. of participants	11,985	1,931	1,910	1,930	1,907		
**All–cause mortality**							
No. of deaths (N=1,506)	890	157	164	152	143		
Model 1	1.00	1.30 (1.09–1.55)	1.40 (1.18–1.66)	1.50 (1.25–1.80)	1.58 (1.25–1.89)	<.0001	1.67 (1.44–1.92)
Model 2	1.00	1.17 (0.96–1.42)	1.26 (1.05–1.51)	1.25 (1.02–1.52)	1.28 (1.05–1.57)	.002	1.30 (1.09–1.56)
Model 3	1.00	1.08 (0.89–1.33)	1.15 (0.95–1.40)	1.14 (0.92–1.42)	1.14 (0.90–1.43)	.120	1.18 (0.96–1.45)
**CVD–mortality**							
No. of deaths (N=487)	277	60	48	47	55		
Model 1	1.00	1.68 (1.24–2.27)	1.34 (0.98–1.83)	1.65 (1.20–2.28)	1.98 (1.46–2.67)	<.0001	1.97 (1.54–2.51)
Model 2	1.00	1.56 (1.12–2.17)	1.27 (0.90–1.79)	1.49 (1.04–2.16)	1.77 (1.24–2.51)	.001	1.72 (1.27–2.32)
Model 3	1.00	1.42 (0.99–2.02)	1.15 (0.80–1.66)	1.38 (0.94–2.02)	1.70 (1.14–2.52)	.018	1.69 (1.18–2.40)
**Cancer mortality**							
No. of deaths (N=410)	273	34	38	35	30		
Model 1	1.00	0.91 (0.63–1.30)	0.98 (0.69–1.39)	1.01 (0.71–1.46)	1.06 (0.72–1.55)	.863	1.03 (0.76–1.38)
Model 2 [e]	1.00	0.81 (0.56–1.18)	0.93 (0.64–1.34)	0.90 (0.61–1.33)	0.93 (0.62–1.42)	.583	0.89 (0.63–1.26)
Model 3 [e]	1.00	0.77 (0.52–1.13)	0.86 (0.58–1.28)	0.86 (0.57–1.31)	0.80 (0.50–1.28)	.407	0.78 (0.52–1.18)

	Processed meat intake (g/d) [b]						
	**Zero intake**	**Quartiles of intake [c]**				**P-trend**	**90th vs 0 [d]**
	**0**	**Q1**	**Q2**	**Q3**	**Q4**		
No. of participants	10,620	2,254	2,293	2,241	2,255		
**All–cause mortality**							
No. of deaths (N=1,506)	779	181	163	185	198		
Model 1	1.00	1.13 (0.96–1.33)	1.36 (1.15–1.63)	1.40 (1.18–1.66)	1.74 (1.48–2.05)	<.0001	1.70 (1.48–1.96)
Model 2	1.00	1.04 (0.88–1.24)	1.21 (0.99–1.47)	1.20 (1.00–1.43)	1.42 (1.18–1.71)	<.001	1.31 (1.12–1.55)
Model 3	1.00	0.98 (0.82–1.18)	1.13 (0.92–1.39)	1.13 (0.92–1.37)	1.33 (1.08–1.65)	.008	1.21 (1.00–1.47)

**CVD–mortality**							
No. of deaths (N=487)	243	66	57	69	52		
Model 1	1.00	1.29 (0.96–1.73)	1.63 (1.21–2.19)	1.89 (1.41–2.52)	1.55 (1.14–2.12)	<.0001	1.72 (1.34–2.22)
Model 2	1.00	1.24 (0.92–1.69)	1.53 (1.09–2.15)	1.67 (1.19–2.35)	1.27 (0.87–1.85)	.015	1.33 (0.97–1.82)
Model 3	1.00	1.16 (0.82–1.63)	1.37 (0.94–1.99)	1.48 (1.01–2.18)	1.07 (0.70–1.62)	.208	1.07 (0.74–1.55)
**Cancer mortality**							
No. of deaths (N=410)	235	40	49	41	45		
Model 1	1.00	0.86 (0.61–1.21)	1.15 (0.81–1.61)	0.96 (0.66–1.40)	1.19 (0.84–1.69)	.442	1.14 (0.83–1.55)
Model 2 [e]	1.00	0.80 (0.56–1.14)	1.07 (0.74–1.53)	0.89 (0.59–1.33)	1.09 (0.74–1.61)	.876	1.00 (0.70–1.43)
Model 3 [e]	1.00	0.76 (0.52–1.11)	1.08 (0.74–1.59)	0.91 (0.59–1.41)	1.09 (0.70–1.71)	.868	0.98 (0.65–1.48)

	Combined intake of red and processed meat (g/d) [b]						
	**Zero intake**		**Quartiles of intake [c]**			**P-trend**	**90th vs 0 [d]**
	**0**	**Q1**	**Q2**	**Q3**	**Q4**		
No. of participants	9,053	2,652	2,653	2,653	2,652		
**All–cause mortality**							
No. of deaths (N=1,506)	665	195	217	219	210		
Model 1	1.00	1.18 (1.00–1.38)	1.25 (1.07–1.47)	1.50 (1.28–1.76)	1.71 (1.46–2.01)	<.0001	1.69 (1.47–1.94)
Model 2	1.00	1.11 (0.93–1.32)	1.15 (0.96–1.38)	1.30 (1.08–1.56)	1.38 (1.14–1.67)	<.001	1.33 (1.12–1.59)
Model 3 [f]	1.00	1.08 (0.90–1.31)	1.12 (0.92–1.36)	1.27 (1.03–1.56)	1.36 (1.09–1.69)	.003	1.32 (1.08–1.60)
**CVD–mortality**							
No. of deaths (N=487)	202	67	83	61	74		
Model 1	1.00	1.37 (1.04–1.82)	1.63 (1.25–2.13)	1.49 (1.11–1.99)	2.04 (1.53–2.72)	<.0001	1.97 (1.55–2.51)
Model 2	1.00	1.41 (1.04–1.90)	1.62 (1.20–2.20)	1.40 (0.99–1.99)	1.82 (1.26–2.64)	.001	1.73 (1.27–2.34)
Model 3 [f]	1.00	1.44 (1.03–2.01)	1.67 (1.19–2.35)	1.44 (0.98–2.12)	1.94 (1.26–2.97)	.005	1.79 (1.26–2.55)
**Cancer mortality**							
No. of deaths (N=410)	207	59	41	59	44		
Model 1	1.00	1.07 (0.80–1.43)	0.75 (0.53–1.05)	1.17 (0.86–1.60)	1.08 (0.77–1.53)	.659	1.05 (0.79–1.39)
Model 2 [e]	1.00	0.99 (0.73–1.36)	0.70 (0.48–1.01)	1.08 (0.76–1.54)	0.93 (0.63–1.37)	.704	0.89 (0.63–1.26)
Model 3 [e f]	1.00	0.92 (0.64–1.32)	0.62 (0.42–0.93)	0.98 (0.65–1.47)	0.81 (0.53–1.23)	.363	0.74 (0.51–1.09)

[a] Data are given as hazard ratio (95% confidence interval).
[b] Values based on energy-adjusted variable.
[c] Quartiles based on percentiles of the energy-adjusted g/d intake among Blacks. Median quartiles (g/d) are as follow: for unprocessed red meat, Q1=4.1, Q2=9.2, Q3=15.6, Q4=42.4; for processed meat, Q1=0.7, Q2=1.5, Q3=3.7, Q4=10; and for combined intake of red and processed meats, Q1=0.8, Q2=6.2, Q3=13.4, Q4=39.3.
Model 1 adjusted for age (attained age as time variable), sex (male and female), and total energy intake (continuous).

Model 2 adjusted for age (attained age as time variable), sex (male and female), total energy intake (continuous), marital status (married/common-law and single/widowed/divorced/separated), educational level (up to high school graduate, trade school/some college/associate degree, bachelor degree, and graduate degree), multivitamin use (current use), smoking status (current smoker, quit <1 year, quit 1–4 years, quit 5–9 years, quit 10–19 years, quit 20–29 years, quit ≥30 years, and never smoked), alcohol use (none, rarely, monthly, weekly, and daily), exercise (none, ≤20 min/wk, 21–60 min/wk, 61–150 min/wk, and ≥151 min/wk), and sleep (≤4 h/night, 5–8 h/night, and ≥9 h/night), body mass index (<18.5, 18.5–24.9, 25.0–29.9, or ≥30.0), aspirin use (used weekly for at least two years in the last five years), having been ever diagnosed with or received treatment in the last 12 months for diabetes, having been diagnosed in the last 5 years with or received treatment in the last 12 months for hypertension or hypercholesterolemia, the use of statin or blood pressure medications for at least 2 years in the last 5 years, and dietary variables (each variable has 5 levels in g/d) as following: cruciferous vegetables (Quintiles: <9.6, 9.6–16.7, >16.7–26.1, >26.1–45.2, >45.2), fruits (Quintiles: <130, 130–224.4, >224.4–322, >322–464.2, >464.2), whole grain (Quintiles: <65, 65–109.9, >109.9–170.3, >170.3–252.2, >252.2), legumes (Quintiles: <17, 17–29.7, >29.7–45.9, >45.9–77.1, >77.1), nuts and seeds (Quintiles: <6.4, 6.4–12.8, >12.8–21.6, >21.6–35.1, >35.1), total dairy (0 intake, quartiles of intake: >0–36, >36–108.1, >108.1–240.9, >240.9), eggs (0 intake, quartiles of intake: >0–3.6, >3.6–7.3, >7.3–20.1, >20.1); and in women, the model also adjusted for menopausal status (premenopausal, postmenopausal), and hormone therapy (in postmenopausal women) (not taking hormone therapy, taking hormone therapy).
Model 3: In addition to covariates in model 2, also adjusted for other meat variables such as fish (0 intake, quartiles of intake: >0–7, >7–12.6, >12.6–21.4, >21.4) and unprocessed poultry (0 intake, quartiles of intake: >0–4.8, >4.8–10.4, >10.4–32.5, >32.5). Also, for model 3 in unprocessed red meat, processed meat was adjusted for (0 intake and quartiles of intake) and vice versa.
[d] Models in theses analyses are correspondents to models 1, 2, and 3, except energy-adjusted log-transformed continuous dietary variables were used instead of five-level adjustment (90th percentile for unprocessed red meat: 43.5 g/d; for processed meat: 11.7 g/d; and for combined intake of red and processed meats: 45.8 g/d).
[e] Also adjusted for previous screening for colon, prostate, or breast cancers during the last four years.
[f] Model 3 here did not adjust for either unprocessed red meat or processed meat, but rather both were combined and used as one exposure variable.

Table S4. The association between red and processed meat intake and all-cause, cardiovascular and cancer mortality among non-Blacks (N=52,486) [a]

	Unprocessed red meat intake (g/d) [b]						
	Zero intake	Quartiles of intake [c]				P-trend	90th vs 0 [d]
	0	Q1	Q2	Q3	Q4		
No. of participants	34,628	4,489	4,476	4,433	4,460		
**All–cause mortality**							
No. of deaths (N=6,455)	4,486	578	514	431	446		
Model 1	1.00	1.13 (1.03–1.24)	1.23 (1.11–1.35)	1.37 (1.24–1.51)	1.64 (1.48–1.81)	<.0001	1.55 (1.44–1.67)
Model 2	1.00	1.06 (0.97–1.17)	1.12 (1.01–1.23)	1.16 (1.04–1.30)	1.27 (1.13–1.42)	<.0001	1.24 (1.13–1.36)
Model 3	1.00	1.05 (0.95–1.17)	1.10 (0.98–1.23)	1.13 (0.99–1.28)	1.21 (1.06–1.38)	.002	1.20 (1.06–1.34)
**CVD–mortality**							
No. of deaths (N=2,111)	1,508	193	161	117	132		
Model 1	1.00	1.15 (0.99–1.35)	1.25 (1.05–1.48)	1.25 (1.03–1.52)	1.62 (1.35–1.94)	<.0001	1.51 (1.31–1.74)
Model 2	1.00	1.11 (0.95–1.31)	1.17 (0.98–1.40)	1.11 (0.90–1.37)	1.33 (1.08–1.62)	.005	1.29 (1.09–1.52)
Model 3	1.00	1.06 (0.89–1.27)	1.09 (0.90–1.33)	1.00 (0.78–1.28)	1.17 (0.92–1.49)	.269	1.17 (0.95–1.43)
**Cancer mortality**							
No. of deaths (N=1,463)	955	141	125	123	119		
Model 1	1.00	1.21 (1.01–1.46)	1.21 (0.99–1.48)	1.54 (1.28–1.87)	1.72 (1.42–2.09)	<.0001	1.68 (1.44–1.96)
Model 2 [e]	1.00	1.10 (0.91–1.33)	1.06 (0.86–1.30)	1.24 (1.01–1.53)	1.24 (0.99–1.54)	.023	1.24 (1.03–1.49)
Model 3 [e]	1.00	1.10 (0.89–1.35)	1.04 (0.82–1.31)	1.20 (0.94–1.52)	1.15 (0.89–1.49)	.159	1.12 (0.89–1.41)
	**Processed meat intake (g/d) [b]**						
	**Zero intake**	**Quartiles of intake [c]**				**P-trend**	**90th vs 0 [d]**
	**0**	**Q1**	**Q2**	**Q3**	**Q4**		
No. of participants	37,507	3,779	3,715	3,795	3,690		
**All–cause mortality**							
No. of deaths (N=6,455)	4,765	470	428	382	410		
Model 1	1.00	1.03 (0.93–1.14)	1.18 (1.06–1.31)	1.24 (1.12–1.39)	1.55 (1.39–1.72)	<.0001	1.47 (1.35–1.60)
Model 2	1.00	0.97 (0.87–1.08)	1.04 (0.93–1.16)	1.06 (0.94–1.20)	1.20 (1.07–1.35)	.005	1.14 (1.03–1.26)
Model 3	1.00	0.93 (0.83–1.05)	0.98 (0.88–1.11)	0.98 (0.86–1.12)	1.09 (0.96–1.24)	.441	1.03 (0.92–1.15)

**CVD–mortality**							
No. of deaths (N=2,111)	1,578	152	141	115	125		
Model 1	1.00	1.06 (0.88–1.28)	1.22 (1.02–1.47)	1.25 (1.02–1.52)	1.55 (1.29–1.88)	<.0001	1.46 (1.25–1.71)
Model 2	1.00	1.01 (0.82–1.24)	1.12 (0.91–1.37)	1.13 (0.92–1.40)	1.33 (1.08–1.63)	.007	1.24 (1.04–1.49)
Model 3	1.00	0.96 (0.77–1.19)	1.06 (0.84–1.33)	1.05 (0.83–1.32)	1.22 (0.97–1.53)	.174	1.10 (0.89–1.36)
**Cancer mortality**							
No. of deaths (N=1,463)	1,059	102	92	97	113		
Model 1	1.00	0.94 (0.76–1.18)	1.09 (0.86–1.38)	1.24 (1.00–1.55)	1.74 (1.43–2.13)	<.0001	1.63 (1.38–1.93)
Model 2 [e]	1.00	0.86 (0.68–1.08)	0.91 (0.71–1.17)	0.99 (0.79–1.24)	1.22 (0.97–1.53)	. 321	1.17 (0.96–1.42)
Model 3 [e]	1.00	0.80 (0.63–1.01)	0.83 (0.65–1.08)	0.87 (0.68–1.12)	1.07 (0.83–1.37)	.773	1.05 (0.84–1.31)

	Combined intake of red and processed meat (g/d) [b]						
	Zero intake	Quartiles of intake [c]				P-trend	90th vs 0 [d]
	0	Q1	Q2	Q3	Q4		
No. of participants	31,234	5,313	5,313	5,313	5,313		
**All–cause mortality**							
No. of deaths (N=6,455)	4,041	687	659	523	545		
Model 1	1.00	1.05 (0.96–1.14)	1.18 (1.08–1.28)	1.30 (1.18–1.43)	1.64 (1.50–1.80)	<.0001	1.52 (1.41–1.64)
Model 2	1.00	1.01 (0.92–1.11)	1.09 (0.99–1.20)	1.13 (1.01–1.25)	1.28 (1.15–1.42)	<.0001	1.22 (1.12–1.34)
Model 3 [f]	1.00	1.00 (0.90–1.11)	1.07 (0.95–1.19)	1.11 (0.98–1.26)	1.25 (1.10–1.42)	<.001	1.20 (1.07–1.35)
**CVD–mortality**							
No. of deaths (N=2,111)	1,362	227	216	149	157		
Model 1	1.00	1.06 (0.90–1.25)	1.21 (1.03–1.41)	1.25 (1.05–1.48)	1.58 (1.33–1.87)	<.0001	1.50 (1.31–1.71)
Model 2	1.00	1.05 (0.87–1.26)	1.15 (0.97–1.36)	1.13 (0.93–1.36)	1.32 (1.08–1.60)	.004	1.30 (1.11–1.53)
Model 3 [f]	1.00	1.01 (0.81–1.25)	1.09 (0.90–1.32)	1.05 (0.84–1.31)	1.23 (0.97–1.55)	.115	1.22 (0.99–1.49)
**Cancer mortality**							
No. of deaths (N=1,463)	873	141	162	134	153		
Model 1	1.00	0.97 (0.80–1.17)	1.23 (1.02–1.47)	1.29 (1.06–1.57)	1.81 (1.52–2.16)	<.0001	1.64 (1.42–1.91)
Model 2 [e]	1.00	0.90 (0.74–1.10)	1.07 (0.88–1.31)	1.04 (0.84–1.28)	1.28 (1.03–1.57)	.051	1.20 (1.00–1.44)
Model 3 [e f]	1.00	0.84 (0.68–1.05)	0.99 (0.79–1.25)	0.96 (0.75–1.23)	1.17 (0.92–1.49)	.261	1.09 (0.86–1.36)

[a] Data are given as hazard ratio (95% confidence interval).

[b] Values based on energy-adjusted variable.

[c] Quartiles based on percentiles of the energy-adjusted g/d intake among non-Blacks. Median quartiles (g/d) are as follow: for unprocessed red meat, Q1=4, Q2=9, Q3=15.6, Q4=41.4; for processed meat, Q1=0.6, Q2=1.4, Q3=3.2, Q4=8.9; and for combined intake of red and processed meats, Q1=2, Q2=8.1, Q3=16.4, Q4=44.4.

Model 1 adjusted for age (attained age as time variable), sex (male and female), and total energy intake (continuous).

Model 2 adjusted for age (attained age as time variable), sex (male and female), total energy intake (continuous), marital status (married/common-law and single/widowed/divorced/separated), educational level (up to high school graduate, trade school/some college/associate degree, bachelor degree, and graduate degree), multivitamin use (current use), smoking status (current smoker, quit <1 year, quit 1–4 years, quit 5–9 years, quit 10–19 years, quit 20–29 years, quit ≥30 years, and never smoked), alcohol use (none, rarely, monthly, weekly, and daily), exercise (none, ≤20 min/wk, 21–60 min/wk, 61–150 min/wk, and ≥151 min/wk), and sleep (≤4 h/night, 5–8 h/night, and ≥9 h/night), body mass index (<18.5, 18.5–24.9, 25.0–29.9, or ≥30.0), aspirin use (used weekly for at least two years in the last five years), having been ever diagnosed with or received treatment in the last 12 months for diabetes, having been diagnosed in the last 5 years with or received treatment in the last 12 months for hypertension or hypercholesterolemia, the use of statin or blood pressure medications for at least 2 years in the last 5 years, and dietary variables (each variable has 5 levels in g/d) as following: cruciferous vegetables (Quintiles: <9.6, 9.6–16.7, >16.7–26.1, >26.1–45.2, >45.2), fruits (Quintiles: <130, 130–224.4, >224.4–322, >322–464.2, >464.2), whole grain (Quintiles: <65, 65–109.9, >109.9–170.3, >170.3–252.2, >252.2), legumes (Quintiles: <17, 17–29.7, >29.7–45.9, >45.9–77.1, >77.1), nuts and seeds (Quintiles: <6.4, 6.4–12.8, >12.8–21.6, >21.6–35.1, >35.1), total dairy (0 intake, quartiles of intake: >0–36, >36–108.1, >108.1–240.9, >240.9), eggs (0 intake, quartiles of intake: >0–3.6, >3.6–7.3, >7.3–20.1, >20.1); and in women, the model also adjusted for menopausal status (premenopausal, postmenopausal), and hormone therapy (in postmenopausal women) (not taking hormone therapy, taking hormone therapy).
Model 3: In addition to covariates in model 2, also adjusted for other meat variables such as fish (0 intake, quartiles of intake: >0–7, >7–12.6, >12.6–21.4, >21.4) and unprocessed poultry (0 intake, quartiles of intake: >0–4.8, >4.8–10.4, >10.4–32.5, >32.5). Also, for model 3 in unprocessed red meat, processed meat was adjusted for (0 intake and quartiles of intake) and vice versa.
[d] Models in theses analyses are correspondents to models 1, 2, and 3, except energy-adjusted log-transformed continuous dietary variables were used instead of five-level adjustment (90th percentile for unprocessed red meat: 47.6 g/d; for processed meat: 10.5 g/d; and for combined intake of red and processed meats: 50.4 g/d).
[e] Also adjusted for previous screening for colon, prostate, or breast cancers during the last four years.
[f] Model 3 here did not adjust for either unprocessed red meat or processed meat, but rather both were combined and used as one exposure variable.

Table S5. The association between red and processed meat intake and all-cause, cardiovascular and cancer mortality among never smokers (N=58,774) [a]

	Unprocessed red meat intake (g/d) [b]						
	Zero intake	Quartiles of intake [c]				P-trend	90th vs 0 [d]
	0	Q1	Q2	Q3	Q4		
No. of participants	39,991	5,055	4,878	4,551	4,299		
**All–cause mortality**							
No. of deaths (N=6268)	4,559	536	451	374	348		
Model 1	1.00	1.12 (1.02–1.24)	1.19 (1.07–1.31)	1.28 (1.15–1.43)	1.54 (1.37–1.72)	<.0001	1.44 (1.32–1.56)
Model 2	1.00	1.06 (0.96–1.18)	1.11 (1.00–1.24)	1.16 (1.02–1.31)	1.34 (1.19–1.52)	<.0001	1.26 (1.14–1.39)
Model 3	1.00	1.04 (0.93–1.16)	1.06 (0.94–1.19)	1.09 (0.95–1.25)	1.22 (1.06–1.41)	.008	1.16 (1.02–1.31)
**CVD–mortality**							
No. of deaths (N=2116)	1,550	189	137	124	116		
Model 1	1.00	1.20 (1.01–1.42)	1.18 (0.99–1.42)	1.32 (1.08–1.62)	1.71 (1.41–2.07)	<.0001	1.55 (1.34–1.78)
Model 2	1.00	1.15 (0.96–1.38)	1.12 (0.93–1.35)	1.20 (0.96–1.49)	1.53 (1.24–1.89)	<.001	1.40 (1.18–1.65)
Model 3	1.00	1.12 (0.92–1.36)	1.06 (0.86–1.29)	1.11 (0.87–1.43)	1.39 (1.09–1.78)	.029	1.26 (1.02–1.56)
**Cancer mortality**							
No. of deaths (N=1401)	1,008	117	107	88	81		
Model 1	1.00	1.04 (0.86–1.27)	1.09 (0.89–1.35)	1.18 (0.95–1.47)	1.34 (1.07–1.69)	.006	1.27 (1.07–1.50)
Model 2 [e]	1.00	0.99 (0.81–1.22)	1.03 (0.83–1.28)	1.06 (0.94–1.35)	1.17 (0.91–1.51)	.268	1.11 (0.91–1.35)
Model 3 [e]	1.00	0.97 (0.78–1.21)	0.98 (0.77–1.25)	1.00 (0.77–1.31)	1.05 (0.79–1.42)	.708	0.99 (0.78–1.27)
	**Processed meat intake (g/d) [b]**						
	Zero intake	Quartiles of intake [c]				P-trend	90th vs 0 [d]
	0	Q1	Q2	Q3	Q4		
No. of participants	41,023	4,454	4,447	4,440	4,410		
**All–cause mortality**							
No. of deaths (N=6268)	4,645	477	394	373	379		
Model 1	1.00	1.06 (0.96–1.17)	1.19 (1.07–1.32)	1.24 (1.10–1.39)	1.44 (1.29–1.62)	<.0001	1.40 (1.28–1.52)
Model 2	1.00	1.02 (0.92–1.14)	1.10 (0.98–1.23)	1.13 (1.00–1.27)	1.30 (1.15–1.47)	<.0001	1.24 (1.12–1.37)
Model 3	1.00	0.99 (0.86–1.12)	1.05 (0.92–1.20)	1.05 (0.92–1.20)	1.18 (1.03–1.35)	.033	1.13 (1.00–1.26)

**CVD–mortality**							
No. of deaths (N=2116)	1,570	164	135	122	125		
Model 1	1.00	1.08 (0.90–1.29)	1.22 (1.01–1.47)	1.42 (1.16–1.74)	1.43 (1.17–1.74)	<.0001	1.46 (1.26–1.70)
Model 2	1.00	1.03 (0.85–1.24)	1.14 (0.93–1.39)	1.32 (1.07–1.62)	1.29 (1.04–1.59)	.001	1.32 (1.11–1.57)
Model 3	1.00	1.00 (0.81–1.23)	1.07 (0.86–1.33)	1.22 (0.97–1.52)	1.13 (0.90–1.43)	.109	1.15 (0.94–1.41)
**Cancer mortality**							
No. of deaths (N=1401)	1,039	94	94	85	89		
Model 1	1.00	0.90 (0.73–1.11)	1.15 (0.92–1.43)	1.07 (0.83–1.37)	1.34 (1.03–1.72)	.030	1.29 (1.07–1.56)
Model 2 [e]	1.00	0.86 (0.70–1.07)	1.06 (0.85–1.33)	0.97 (0.74–1.26)	1.22 (0.93–1.60)	.343	1.16 (0.94–1.43)
Model 3 [e]	1.00	0.83 (0.66–1.04)	1.02 (0.80–1.30)	0.92 (0.69–1.23)	1.13 (0.84–1.52)	.752	1.10 (0.86–1.40)

Combined intake of red and processed meat (g/d) [b]

	Zero intake	Quartiles of intake [c]				P-trend	90th vs 0 [d]
	**0**	**Q1**	**Q2**	**Q3**	**Q4**		
No. of participants	34,912	5,965	5,966	5,965	5,966		
**All–cause mortality**							
No. of deaths (N=6268)	4,041	620	613	504	490		
Model 1	1.00	1.06 (0.96–1.16)	1.12 (1.02–1.22)	1.23 (1.12–1.36)	1.50 (1.36–1.65)	<.0001	1.44 (1.33–1.56)
Model 2	1.00	1.04 (0.95–1.15)	1.06 (0.97–1.17)	1.14 (1.02–1.26)	1.31 (1.17–1.47)	<.0001	1.26 (1.15–1.39)
Model 3 [f]	1.00	1.03 (0.93–1.14)	1.04 (0.93–1.16)	1.11 (0.98–1.25)	1.27 (1.11–1.45)	.002	1.22 (1.08–1.37)
**CVD–mortality**							
No. of deaths (N=2116)	1,376	210	216	158	156		
Model 1	1.00	1.04 (0.89–1.22)	1.22 (1.05–1.41)	1.27 (1.08–1.51)	1.54 (1.30–1.84)	<.0001	1.52 (1.33–1.74)
Model 2	1.00	1.03 (0.87–1.21)	1.17 (0.99–1.38)	1.16 (0.97–1.39)	1.38 (1.13–1.68)	<.001	1.40 (1.18–1.65)
Model 3 [f]	1.00	1.02 (0.84–1.22)	1.15 (0.95–1.38)	1.12 (0.90–1.39)	1.30 (1.03–1.66)	.032	1.34 (1.08–1.65)
**Cancer mortality**							
No. of deaths (N=1401)	899	133	131	119	119		
Model 1	1.00	0.98 (0.80–1.21)	1.01 (0.84–1.22)	1.05 (0.84–1.32)	1.39 (1.15–1.69)	.011	1.25 (1.06–1.47)
Model 2 [e]	1.00	0.96 (0.78–1.19)	0.95 (0.78–1.16)	0.96 (0.76–1.23)	1.21 (0.97–1.51)	.381	1.10 (0.90–1.33)
Model 3 [e f]	1.00	0.92 (0.73–1.16)	0.90 (0.72–1.12)	0.91 (0.69–1.20)	1.12 (0.87–1.45)	.789	1.00 (0.78–1.27)

[a] Data are given as hazard ratio (95% confidence interval).

[b] Values based on energy-adjusted variable.

[c] Quartiles based on percentiles of the energy-adjusted g/d intake among never-smoked participants. Median quartiles (g/d) are as follow: for unprocessed red meat, Q1=4, Q2=9, Q3=15.6, Q4=41.1; for processed meat, Q1=0.6, Q2=1.3, Q3=2.9, Q4=8; and for combined intake of red and processed meats, Q1=1.1, Q2=6.6, Q3=13.9, Q4=38.9.

Model 1 adjusted for age (attained age as time variable), sex (male and female), race (black, nonblack), and total energy intake (continuous).

Model 2 adjusted for age (attained age as time variable), sex (male and female), race (black and nonblack), total energy intake (continuous), marital status (married/common-law and single/widowed/divorced/separated), educational level (up to high school graduate, trade school/some college/associate degree, bachelor degree, and graduate degree), multivitamin use (current use), alcohol use (none, rarely, monthly, weekly, and daily), exercise (none, ≤20 min/wk, 21–60 min/wk, 61–150 min/wk, and ≥151 min/wk), and sleep (≤4 h/night, 5–8 h/night, and ≥9 h/night), body mass index (<18.5, 18.5–24.9, 25.0–29.9, or ≥30.0), aspirin use (used weekly for at least two years in the last five years), having been ever diagnosed with or received treatment in the last 12 months for diabetes, having been diagnosed in the last 5 years with or received treatment in the last 12 months for hypertension or hypercholesterolemia, the use of statin or blood pressure medications for at least 2 years in the last 5 years, and dietary variables (each variable has 5 levels in g/d) as following: cruciferous vegetables (Quintiles: <9.6, 9.6–16.7, >16.7–26.1, >26.1–45.2, >45.2), fruits (Quintiles: <130, 130–224.4, >224.4–322, >322–464.2, >464.2), whole grain (Quintiles: <65, 65–109.9, >109.9–170.3, >170.3–252.2, >252.2), legumes (Quintiles: <17, 17–29.7, >29.7–45.9, >45.9–77.1, >77.1), nuts and seeds (Quintiles: <6.4, 6.4–12.8, >12.8–21.6, >21.6–35.1, >35.1), total dairy (0 intake, quartiles of intake: >0–36, >36–108.1, >108.1–240.9, >240.9), eggs (0 intake, quartiles of intake: >0–3.6, >3.6–7.3, >7.3–20.1, >20.1); and in women, the model also adjusted for menopausal status (premenopausal, postmenopausal), and hormone therapy (in postmenopausal women) (not taking hormone therapy, taking hormone therapy).

Model 3: In addition to covariates in model 2, also adjusted for other meat variables such as fish (0 intake, quartiles of intake: >0–7, >7–12.6, >12.6–21.4, >21.4) and unprocessed poultry (0 intake, quartiles of intake: >0–4.8, >4.8–10.4, >10.4–32.5, >32.5). Also, for model 3 in unprocessed red meat, processed meat was adjusted for (0 intake and quartiles of intake) and vice versa.

[d] Models in theses analyses are correspondents to models 1, 2, and 3, except energy-adjusted log-transformed continuous dietary variables were used instead of five-level adjustment (90th percentile for unprocessed red meat: 43.9 g/d; for processed meat: 9.4 g/d; and for combined intake of red and processed meats: 44.6 g/d).

[e] Also adjusted for previous screening for colon, prostate, or breast cancers during the last four years.

[f] Model 3 here did not adjust for either unprocessed red meat or processed meat, but rather both were combined and used as one exposure variable

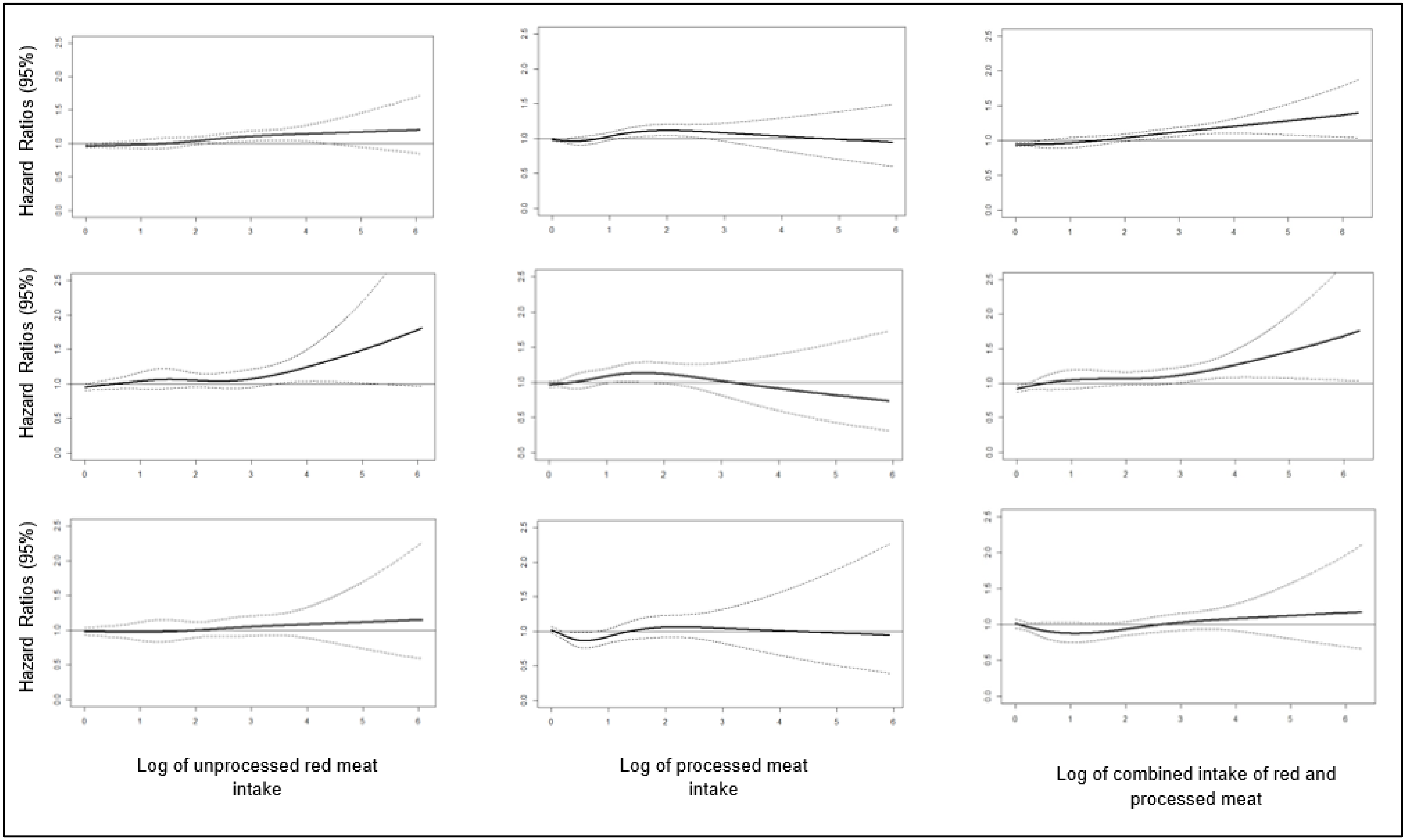

Figure S1. Dose-response relationships of red and processed meats with the risk of all-cause (top row), CVD (middle row) and cancer (bottom row) mortality in the AHS-2 cohort.

The spline models were adjusted for age (attained age as time variable); sex (male, female); race (blacks, non-blacks); marital status (married/common-law, single/widowed/divorced/separated); education level (up to high school graduate, trade school/some college/associate degree, bachelor degree, graduate degree); multivitamin use (current use, past or never used); smoking (current smoker, quit <1 year, quit 1–4 years, quit 5–9 years, quit 10–19 years, quit 20–29 years, quit ≥30 years, never smoked); alcohol use (none, rarely, monthly, weekly, daily); exercise (none, ≤20 min/week, 21–60 min/week, 61–150 min/week, ≥151 min/week); sleep (≤4 hours/night, 5–8 hours /night, ≥9 hours /night); body mass index (BMI) (<18.5, 18.5–24.9, 25.0–29.9, ≥30.0); diabetes mellitus (yes/no: having ever been diagnosed with or received treatment in the last 12 months for diabetes); hypertension (yes/no: having been diagnosed in the last 5 years with or received treatment in the last 12 months for hypertension); hypercholesterolemia (yes/no: having been diagnosed in the last 5 years with or received treatment in the last 12 months for hypercholesterolemia); aspirin use (yes/no: used weekly for at least two years

in the last five years); the use of blood pressure medications for at least 2 years in the last 5 years (yes/no); the use of statin for at least 2 years in the last 5 years (yes/no);— and in women, menopausal status among women (premenopausal, postmenopausal); hormone replacement therapy (HRT) among postmenopausal women (yes/no: taking HRT)—; dietary energy (continuous); and dietary variables (continuous) including cruciferous vegetables, fruits, whole grain, legumes, nuts and seeds, total dairy, eggs, fish, unprocessed poultry, and mutual adjustment of unprocessed red meat and processed meat. For combined intake of both unprocessed red and processed meats, both were summed together and used as one exposure variable.
